# On the Ability of Low Molecular Weight Chitosan Enzymatically Depolymerized to Produce and Stabilize Silver Nanoparticles

**DOI:** 10.3390/biomimetics3030021

**Published:** 2018-08-13

**Authors:** Inmaculada Aranaz, Carolina Castro, Angeles Heras, Niuris Acosta

**Affiliations:** 1 Department of Chemistry in Pharmaceutical Sciences, Pharmacy Faculty, Complutense University, Plaza de Ramón y Cajal, s/n, 28040 Madrid, Spain; aheras@ucm.es; 2 Biofunctional Studies Institute, Complutense University, Paseo Juan XXIII, 1, 28040 Madrid, Spain; caroca@ucm.es; 3 Sustainable Physical-Chemical Innovation, S.L. (InFiQus), 28040 Madrid, Spain

**Keywords:** quantum dots, metallic nanoparticles, green synthesis, silver nanoparticles, natural polymers, chitosan, functional characterization, lysozyme, chitosanase

## Abstract

Silver nanoparticles (AgNPs) are of great interest due to their antimicrobial, optical and catalytical properties. Green synthesis of AgNPs is fundamental for some applications such as biomedicine and catalysis. Natural polymers, such as chitosan, have been proposed as reducing and stabilizing agents in the green synthesis of AgNPs. Physico-chemical properties of chitosan have a great impact on its technological and biological properties. In this paper, we explore the effect of chitosan molecular weight (Mw) on the thermal AgNPs production using two sample sets of low Mw chitosans (F1 > 30 kDa, F2: 30–10 kDa and F3: 10–5 kDa) produced by enzymatic depolymerization of a parent chitosan with chitosanase and lysozyme. Both polymer sets were able to effectively reduce Ag^+^ to Ag^0^ as the presence of the silver surface plasmon resonance (SRP) demonstrated. However, the ability to stabilize the nanoparticles depended not only on the Mw of the polymer but particularly on the polymer pattern which was determined by the enzyme used to depolymerize the parent chitosan. Low Mw chitosan samples produced by lysozyme were more effective than those produced by chitosanase to stabilize the AgNPs and smaller and less polydisperse nanoparticles were visualized by transmission electron microscopy (TEM). With some polymer sets, more than 80% of the AgNPs produced were lower than 10 nm which correspond to quantum dots. The preparation method described in this paper is general and therefore, it may be extended to other noble metals, such as palladium, gold or platinum.

## 1. Introduction

Metallic nanoparticles (NPs) (e.g., platinum, silver, gold, etc.) are of interest due to their unique optical, electrical, biomedical, or chemical properties among others [1]. Different preparation methods have been reported in the literature to produce metallic nanoparticles such as conventional chemical reduction, radiation chemical reduction, or sonochemical reduction [2,3,4,5,6]. Chemical reduction is generally carried out with non-eco-friendly and/or toxic agents such as NaBH_4_ which hindered their application in some fields such as biomedicine or fine catalysis. On the other hand, the physical methods avoid these toxic products but they require the use of expensive technologies. Therefore, there is a need to synthesize nanoparticles using eco-friendly and clean processes. Several methods to produce silver nanoparticles assisted with microorganisms and natural products such as plant extracts or polymers such as chitosan, starch, agar, chondroitin sulfate, or heparin have been reported [1,7,8,9,10,11]. In general, silver nanoparticles (AgNPs) produced with these natural products are polydisperse and their sizes range between 5 and 200 nm. Natural polymers are not only able to reduce Ag^+^ to Ag^0^ but they also are able to stabilize the nanoparticles avoiding their aggregation. Among natural polymers, chitosan is a very promising molecule due to its technological and biological properties. Chitosan is a copolymer composed by *N*-acetylglucosamine (GlcNAc, A monomer) and glucosamine (GlcN, D monomer) in variable proportion. Chitosan biological and technological properties depend on the physicochemical characteristics of the polymer, mainly its acetylation degree, Mw, and polymer pattern [12]. Chitosan has been used both as reducing and stabilizing agent in the green synthesis of metallic nanoparticles, and some influence of the polymer physicochemical properties on the characteristics of the nanoparticles could be inferred although a poor physicochemical characterization of the polymers was carried out [12]. In Table 1, some examples of AgNPs produced by different methodologies using chitosan as stabilizing or reducing/stabilizing agent are depicted. Low Mw chitosan samples have been related to low size AgNPs and lower polydispersity but since some methods used for AgNPs production also reduce chitosan Mw (i.e., sonochemical or radiation methods), the comparison is not trivial.

Low Mw chitosan samples can be produced by chitosan depolymerization using different methodologies such as chemical depolymerization, radiation, sonolysis, or enzymatic depolymerization [12]. In the particular case of enzymatic depolymerization, depending on the chosen enzyme, the samples exhibit different patterns. That is, the distribution of the monomeric units (*N*-acetylglucosamine and glucosamine) along the polymer chains differs depending on the selected enzyme. Previous studies have shown pronounced differences between the low Mw chitosan samples generated either by lysozyme or chitosanase [18].

The aim of this paper is to evaluate the ability of low Mw chitosan samples produced by enzymatic degradation to reduce Ag^+^ to Ag^0^ and to stabilize the AgNPs. Our hypothesis is that not only chitosan Mw or acetylation degree but also the polymer pattern plays a fundamental role in AgNPs production and stabilization. We have depolymerized chitosan with lysozyme and chitosanase to produce low Mw chitosan samples with different patterns and the ability of these polymers to produce and stabilize AgNPs has been evaluated. The growth of nanoparticles has been monitored by ultraviolet–visible (UV–Vis) spectroscopy and the formed nanoparticles have been characterized by transmission electron microscopy (TEM).

## 2. Materials and Methods

Chitosan was provided by InFiQus, S.L. (Madrid, Spain), silver nitrate (purity ≥ 99%), lysozyme from chicken hen (EC 3.2.1.17) and chitosanase from *Streptomyces griseus* (EC 3.2.1.132) were provided by Sigma-Aldrich (St. Louis, MO, USA), dialysis membranes (MWCO-3500, 7000 or 12–14,000 Da) were provided by Medicell International, Ltd. (London, UK). Milli-Q water was used in all experiments.

### 2.1. Enzymatic Chitosan Depolymerization

Chitosan was dissolved in either 0.2 M acetic/acetate buffer at 0.5% (*w*/*v*) (pH 5.7) or in 0.2 M acetic/acetate buffer at 0.5% (*w*/*v*) (pH 4.5) and depolymerized using a commercial chitosanase or lysozyme, respectively. One milliliter of enzyme (3.48 × 10^−3^ mg/mL and 62.7 × 10^−2^ mg/mL, respectively) was employed per 10 mL or 100 mL of substrate (lysozyme reaction or chitosanase reaction) and the reaction was carried out at 37 °C in an orbital Lab Therm LT-X shaker (Thermo Fisher Scientific, Inc., Waltman, MA, USA) at 100 rpm for four days (chitosanase) and seven days (lysozyme) [18].

The depolymerized chitosans were separated by tangential ultrafiltration using Vivaflow Crossflow Cassettes connected to a Vivaflow 200 system (Sartorius-Stedim Biotech, Göttingen, Germany) with 30, 10, and 5 kDa cut-off polyethersulfone membranes. Three fractions were isolated from each process: fraction F1 (Mw > 30 kDa), fraction F2 (Mw 30–10 kDa) and fraction F3 (Mw 10–5 kDa). Fractions were dialyzed against distilled water in membranes with Mw 12–14, 7 and 3.5 kDa cut-off, respectively, until complete salt elimination. Dialyzed fractions were freeze-dried and a white powder was recovered.

### 2.2. Polymer Characterization

Polymer acetylation degree (F_A_) was determined by the first derivative UV spectrophotometric method using a Specord 205 spectrophotometer (Analtykjena, Jena, Germany) [19]. Polymer Mw and polydispersity index (PDI) were determined by size exclusion-high-performance liquid chromatography (SEC-HPLC). Size exclusion chromatography was carried out in a Waters 625 LC System Pump with an Ultrahydrogel column (I.D.: 7.8 mm, L: 300 mm) thermostated at 35 °C and connected to a Waters refraction index detector (Waters, Corp., Milford, MA, USA). Samples were dissolved at 3 mg/mL in the eluent (0.2 M acetic acid/0.15 M ammonium acetate buffer (pH 4.5)). Dextran standard kit (WAT054392, Waters, Corp.) was used to produce a standard curve to determine the average Mw of the chitosan samples.

### 2.3. Silver Nanoparticles Production and Characterization

Parent chitosan was dissolved in acetic acid 0.1 M and depolymerized chitosan samples were dissolved in distilled water at a polymer concentration of 1% *w*/*v* under magnetic stirring (150 rpm) overnight. The pH of parent chitosan dissolved in acetic acid was 3.6 while the pH of the depolymerized chitosan samples dissolved in water ranged between 6.2 and 6.5 (pH-meter GLP 21, electrode 52-02, Crison Instruments, L’Hospitalet de Llobregat, Spain). To one milliliter of each polymeric solution, 200 µL of AgNO_3_ (5 mM) dissolved in water were added, the mixture was vortexed for 30 s. Finally, the solutions were heated at 90 °C for 5 h without stirring.

The formation of AgNPs within the chitosan solutions was followed by the appearance of the characteristic Ag exciton peak around 400 nm. Transmission electronic microscopy was used to determine AgNPs size and morphology. Micrographs were analyzed with ImageJ (National Institutes of Health, USA) and Origin (OriginLab, Northampton, MA, USA) software’s to produce particles histograms (at least 50 nanoparticles per sample were analyzed). The crystalline nature of the AgNPs was determined by selected area electron diffraction (SAED) and X-ray diffraction (XRD) (Malvern Panalytical’s X-ray diffractometer, Philips model, Malvern, UK). Silver nanoparticles particle size and zeta potential were determined by dynamic light scattering (DLS) and laser doppler electrophoresis, respectively. Determinations were carried out in triplicates in a Nano-Zetasizer system (Malvern Panalytical).

## 3. Results

### 3.1. Polymer Characterization

Parent chitosan sample was enzymatically depolymerized using chitosanase and lysozyme and the resulting products were fractionated into three samples; fraction F1 (Mw > 30 kDa), fraction F2 (Mw 30–10 kDa), and fraction F3 (Mw 10–5 kDa). All samples were characterized in terms of their physicochemical properties (polymer acetylation degree, average Mw, and polydispersity index) as shown in Table 2. The depolymerization slightly reduced the acetylation degree of the polymer fractions, with this effect being more remarkable in the chitosanase fractions. In all cases, the size polydispersity of the polymer fractions was lower than the parent chitosan. When comparing fractions isolated with the same membrane similar Mw were observed.

### 3.2. Silver Nanoparticle Production

**Figure 1:**
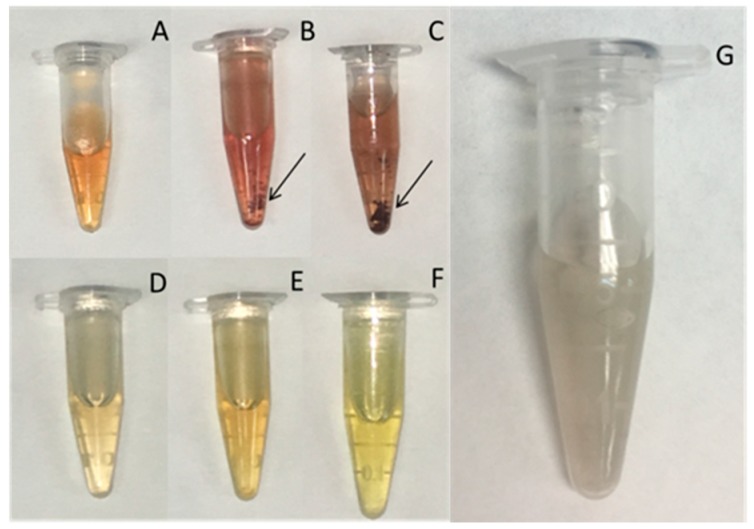
Visual evaluation of AgNp-polymer solutions after 5 h at 90 °C (day 0). (**A**) F1Q, (**B**) F2Q, (**C**) F3Q, (**D**) F1L, (**E**) F2L, (**F**) F3L, and (**G**) parent chitosan. Arrows indicate the presence of aggregates.

The polymeric solutions containing AgNO_3_ were heated at 90 °C for 5 h. The color of the solutions changed from colorless to light yellow (FiL fractions) or yellowish brown (FiQ fractions). The AgNO_3_ solution of the control sample (parent chitosan) changed to a greyish color. The color change confirmed the synthesis of AgNPs (Figure 1). Silver nanoparticles synthesized in the presence of F2Q and F3Q samples tended to form large aggregates with time and in 24 h they were observed by the naked eye.

**Figure 2:**
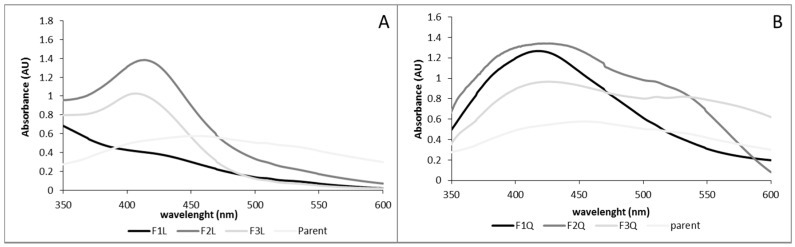
Ultraviolet–visible (UV–Vis) spectra of AgNPs synthesized using different low molecular weight chitosan fractions as reducing and stabilizing agent. (**A**) Chitosan depolymerized using chitosanase and (**B**) chitosan depolymerized using lysozyme. In both figures, the UV–Vis spectrum of the parent chitosan has been added for comparison.

Ultraviolet spectra of the fresh solutions are depicted in Figure 2. The characteristic surface plasmon resonance (SPR) peak at around 400 nm was observed in the samples confirming the nanoparticle formation [20]. Silver nanoparticles produced by the parent chitosan showed a broad spectrum with a maximum absorbance of around 450 nm. Silver nanoparticles produced with the assistance of chitosan depolymerized with chitosanase showed broad spectra with a maximum absorbance around 417–423 nm. Silver nanoparticles produced with the assistance of chitosan depolymerized with lysozyme exhibited narrower spectra with a maximum around 403–409 nm when F2L and F3L were studied. When F1L was used to produce the AgNPs a weak and broad SPR occurred. It is well known that the characteristics of the AgNPs spectra depend on the AgNPs properties (i.e., size and polydispersity). Blue shifts of λ_max_ have been reported as the size of the AgNPs is reduced [21]. Moreover, the shape of the spectrum is related to sample polydispersity, a broad spectrum indicates broad AgNPs distribution. The blue shift of the λ_max_, when depolymerized fractions were tested indicated that the low Mw chitosan fractions produced lower sized AgNPs than the parent chitosan. Moreover, the narrower spectra, in particular when FiL were used to produce the nanoparticles, indicated the presence of less polydisperse nanoparticles.

### 3.3. Nanoparticles Characterization

The AgNPs were characterized in terms of charge and size (Table 3). The charge of AgNPs was evaluated by determining their zeta potential. In all cases, positive values ranging from +10 to +40 mV were observed. Similar zeta potential values were observed when similar fractions were compared so this parameter did not allow us to predict AgNPs stability. The average size of the nanoparticles determined by TEM ranged from 7 to 200 nm while large sizes were determined by DLS due to polymer capping. During this initial characterization, AgNPs produced with the fractions F2Q and F3Q revealed low stability forming large aggregates and were discharged for further characterization.

**Figure 3:**
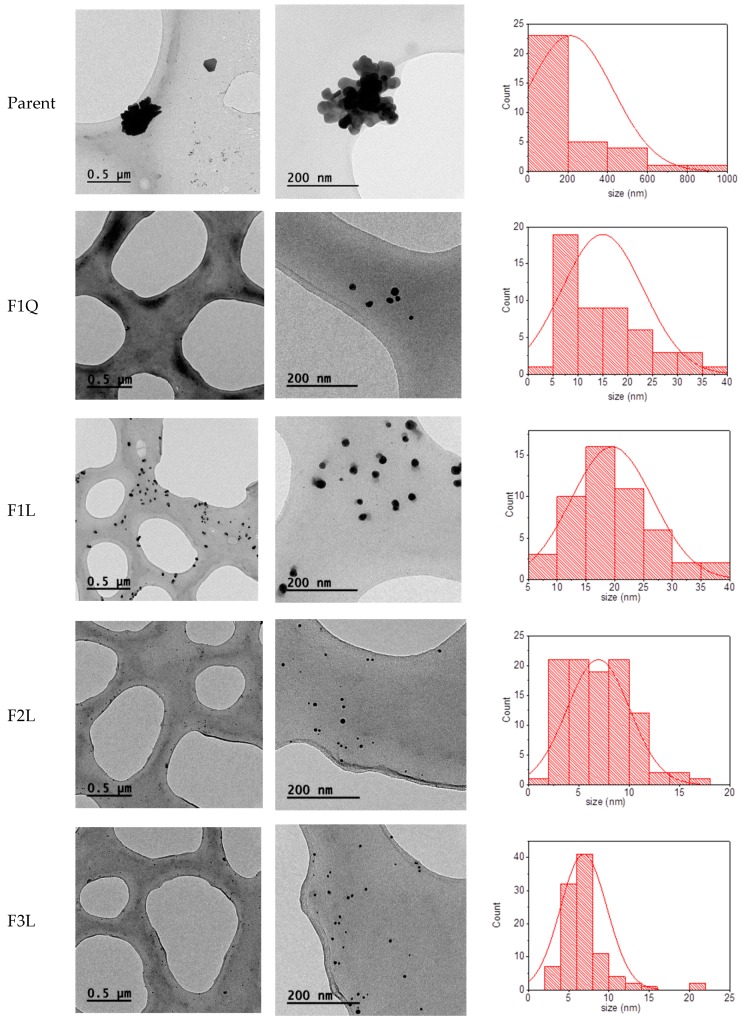
Transmission electron microscopy (TEM) micrographs and size histograms based on TEM images.

The TEM provided further insight into the morphology and size details of the synthesized AgNPs. The images at different magnifications are shown in Figure 3. In all cases, nanoparticles produced using low Mw chitosans were mainly spherical in shape and they were well dispersed. In contrast, some triangles, spheres, and well-dispersed clusters were observed in the parent sample. When fractions F1Q and F1S were used to produce the AgNPs larger and more polydisperse nanoparticles were observed which is in agreement with the observed spectra in Figure 2. On the contrary, fraction F2L and fraction F3L produced smaller nanoparticles with lower dispersity, in particular, F3L showed a narrow particle size distribution which is in good agreement with the UV–Vis spectrum showed in Figure 2.

**Figure 4:**
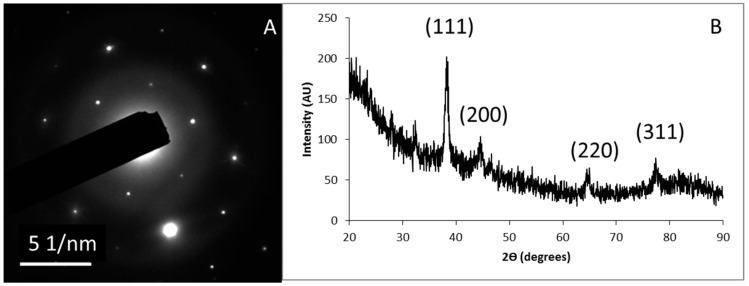
Crystalline nature of AgNPs. (**A**) Selected area electron diffraction (SAED) pattern of AgNPs produced with F2L and (**B**) X-ray diffraction (XRD) pattern.

An example of a SAED pattern of one of the nanoparticles sets is shown in Figure 4A. Concentric rings were observed which corresponds to the different crystalline planes of the synthesized AgNPs. The crystalline nature of the AgNPs was further confirmed by their XRD pattern (Figure 4B), the main characteristic diffraction peaks for silver are observed at 2θ = 38.22°, 44.62°, 64.54° and 77.42° with correspond to crystallographic planes of (111), (200), (220), and (311), respectively. By comparing with JCPDS (Joint Committee on Powder Diffraction Standards, file no.: 89-3722), the synthesized AgNPs were found to possess a face centered cubic (fcc) structure.

### 3.4. Nanoparticles Stability

**Figure 5:**
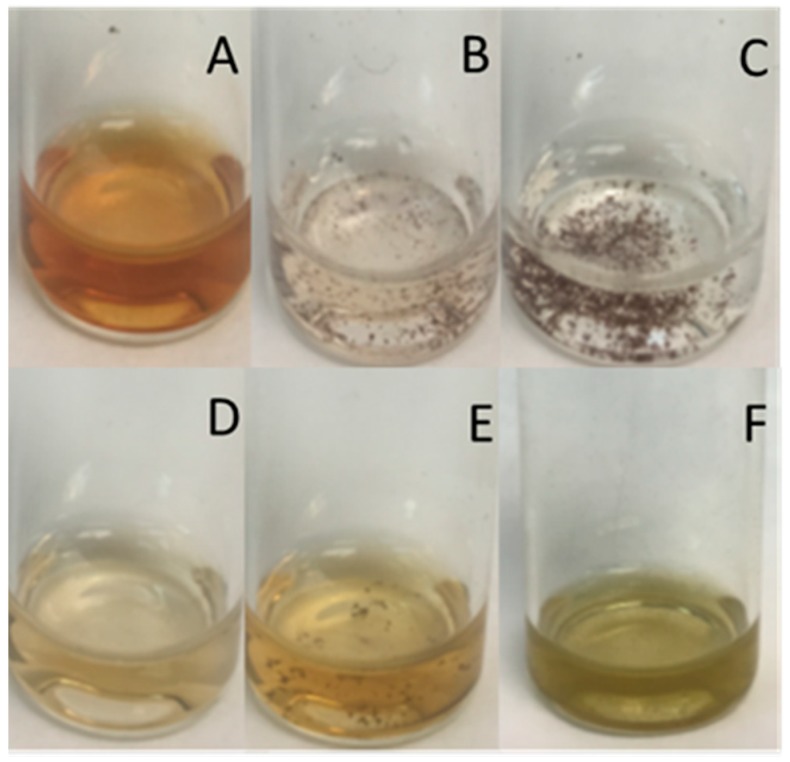
Visual evaluation of AgNP-polymer solutions after 5 h at 90 °C (one month). (**A**) F1Q, (**B**) F2Q, (**C**) F3Q, (**D**) F1L, (**E**) F2L, and (**F**) F3L.

The stability of the nanoparticles in dark at room temperature was evaluated after one month. According to the previous results, AgNPs produced with chitosan fractions F2Q and F3Q were completely aggregated and almost uncolored solutions were observed. In fractions F1Q and F1L transparent solutions were observed and in fractions F2L and F3L some sedimented particles were observed; these sedimented particles were easily resuspended by gentle agitation but the they settled again in less than 60 min (Figure 5).

**Figure 6:**
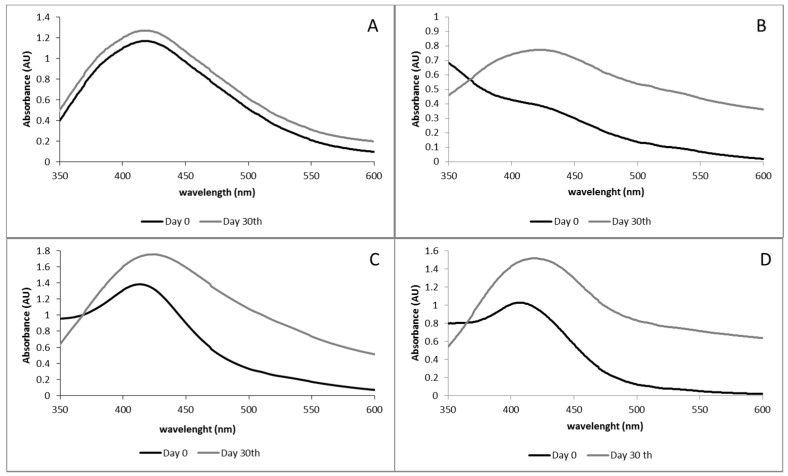
Ultraviolet–visible spectra of AgNPs synthesized using different low molecular weight chitosan fractions as reducing and stabilizing agent. (**A**) F1Q, (**B**) F1L, (**C**) F2L, and (**D**) F3L.

Ultraviolet–visible spectroscopy can be used as a simple method for monitoring the stability of nanoparticle solutions. Therefore, the SRP of the nanoparticles was also measured and compared to the one of the fresh AgNPs (Figure 6). In all cases, at 30 days an increase of the absorbance intensity was observed. Silver nanoparticles produced with fraction F1Q maintained the λ_max_ and the spectra shape which indicated that this fraction was particularly effective in the stabilization of the AgNPs. The spectra of fraction F1L was narrower and more intense, probably because AgNPs needed some time after thermal exposure to properly form. Finally, AgNPs spectra of fraction F2L and F3L shifted from 413 to 420 nm and from 408 to 416 nm, respectively. The red-shift and the presence of absorbance at λ > 500 nm can be explained due to the presence of aggregates in good agreement with the visual exploration of the solutions.

## 4. Discussion

Silver nanoparticle synthesis occurs in a multi-step process; firstly Ag^+^ is reduced to Ag^0^ with the assistance of a reducing agent. Secondly, silver atoms (Ag^0^) agglomerate into clusters and finally colloidal AgNPs are formed. Due to the tendency of AgNPs to aggregate, the use of stabilizing agents is mandatory to produce small nanoparticles.

It has been described that chitosan can served simultaneously as a reducing agent for silver cation and a stabilizing agent for AgNPs. In this paper, we have evaluated the ability of a series of low Mw chitosan samples to reduce silver ions to silver atoms and to stabilize the formed AgNPs. A chitosan sample was depolymerized with chitosanase or lysozyme and the reaction mixture was separated into three fractions. As seen in Table 2, little differences between fractions produced by each enzyme were observed regarding Mw and polydispersity. Due to enzyme specificity, samples with similar Mw will differ in their patterns depending on the enzyme used to depolymerize the parent chitosan. For instance, lysozyme hydrolyzes chitosan by cleavage of glycosidic bonds of the type –AA|AA– and –AA|AD–, whereas –AD|AA– and –DD|AA– are not, or only very slowly, susceptible to hydrolysis. On the contrary, chitosanase hydrolyzes chitosan by cleavage of glycosidic bonds of the type –DD|DA–, –DD|DD– (where A denoted *N*-acetylglucosamine and D denoted glucosamine units, respectively) [22]. A previous study revealed the different patters achieved by using lysozyme or chitosanase to hydrolyze a parent chitosan (FA 0.25). The sample hydrolyzed with lysozyme was mainly composed by hetero-copolymers while the sample hydrolyzed with chitosanase was mainly composed by fully deacetylated oligomers of GlcN as well as mono-, di-, and triacetylated homologs of the molecular composition (D_n − m_A_m_, n = 4–24, m = 0–3, n + m < 25) [18]. As observed in Table 2, the depolymerization reduced the acetylation degree in all sample, especially this reduction was more remarkable in FiQ fractions. The reduction of the acetylation degree should not affect the ability of the polymer to stabilize AgNPs since previous studies have shown that AgNPs are produced and stabilized by chitosan samples within a large range of acetylation degrees [13,14,15,16].

The presence of the silver exciton in all samples (parent chitosan and depolymerized fractions) indicates that all fractions were able to effectively reduce Ag^+^ to Ag^0^ no matter their physico-chemical properties. However, chitosanase fractions were less effective to stabilize the nanoparticles than the lysozyme ones. In fact, only the nanoparticles produced with the fraction F1Q (>30 kDa) were stable while those produced with the lower Mw fractions (F2Q and F3Q) tended to aggregate forming large clusters observed by the naked eye.

On the contrary, AgNPs formed in the presence of chitosan fractions depolymerized by lysozyme showed a good ability to stabilize the nanoparticles. In fact, fraction F3L was able to stabilize the nanoparticles with a small size (lower than 10 nm) and monodisperse nanoparticles were observed. Interestingly, in long-term experiments, F1Q was the most effective fraction regarding nanoparticles stabilization since the SPR was almost unaltered after 30 days.

It is generally accepted that chitosan Mw and acetylation degree affect the AgNPs formation although a systematic study has not been carried out. Moreover, due to poor or inexistent polymer characterization, it is not trivial to determine how these properties affect AgNPs formation. Results point that medium and low Mw polymers are more effective to stabilize AgNPs and some authors point to the role of primary amino groups in the AgNPs stabilization [12]. Some studies have revealed that not only chitosan Mw or acetylation degree but also its pattern—that is, how *N*-acetylglucosamine and glucosamine units are distributed along the polymer chain—have effect on chitosan technological and biological properties [12,18,23].

When fractions F1Q and F1L are compared strong differences regarding size, polydispersity, and stability are observed. These fractions are quite similar regarding Mw and acetylation degree; therefore, we consider that the strong differences, regarding AgNPs production and stabilization found between these samples are likely due to the different polymer pattern. Samples F2Q and F2L as well as F3Q and F3L exhibited similar Mw but different acetylation degrees so differences cannot be ascribed solely to polymer pattern. However, it is very remarkable the lack of ability of samples F2Q and F3Q to stabilize the AgNPs and the ability of other chitosan samples with similar acetylation degrees to stabilize AgNPs [15].

## 5. Conclusions

This work has demonstrated that AgNPs size and stability can be tailored by selecting the appropriate chitosan sample. These results indicate that not only chitosan acetylation degree or Mw as previously reported have a strong influence on the green AgNPs synthesis and stabilization. Polymer pattern also plays a fundamental role on AgNPs synthesis and stabilization. Taking into account that a large number of depolymerization methods are available and that the physicochemical properties of the obtained depolymerized samples will depend on both the physicochemical properties of the parent chitosan and the depolymerization method it is necessary to carry out systematic research on this issue.

Further research must be carried out to study how to balance mixtures of chitosan with different physicochemical properties in order to produce AgNPs for specific purposes. Moreover, thermal reduction of noble metal ions in the presence of natural polymers to produce metallic nanoparticles is a general method; therefore, these findings are likely to be useful in the green synthesis of other metallic nanoparticles.

## Figures and Tables

**Table 1:** Physico-chemical properties of the chitosan samples used in the synthesis of silver nanoparticles (AgNPs) and their main characteristics.

Polymer Characteristics			AgNPs Characteristics		
Mw (kDa)	FA	Dispersion	Shape	Size (nm)	Ref.
1240	0.13	Polydisperse	Spherical	10–150	[13]
400	0	Polydisperse	Spherical, Clusters	20–100	[14]
High Mw	0.25	Monodisperse	Spherical	4–8	[15]
High Mw	0.15	Polydisperse	Spherical, Fractal	20–200	[16]
-	-	Monodisperse	Spherical	4–5	[6]
-	-	Polydisperse	Spherical	10–80	[17]

Mw: Molecular weight; FA: Fraction of acetylated units.

**Table 2:** Physico-chemical characterization of polymer samples.

Sample ^1^	FA	Mn (kDa)	Mw (kDa)	PDI
F1Q	38.54 ± 0.78	16,048	42,196	2.63
F2Q	31.07 ± 0.71	6207	10,312	1.66
F3Q	26.03 ± 0.27	5123	7020	1.37
F1L	38.43 ± 1.48	11,768	30,933	2.63
F2L	42.29 ± 1.63	5793	8171	1.41
F3L	42.67 ± 1.18	3578	4232	1.18
Parent	48.34 ± 1.83	128,008	538,448	4.21

^1^ FiQ: Fractions obtained by depolymerization with chitosanase; FiL: Fractions obtained by depolymerization with lysozyme. FA: Fraction of acetylated units; Mn: Number average molecular weight; Mw: Molecular weight; PDI: Polydispersity index.

**Table 3:** AgNPs characterization.

Sample ^1^	Zeta Potential (mV)	Size (nm) ^2^	Size (nm) ^3^	<10 nm (%)
F1Q	+24.0 ± 6.5	202.5	15	40
F2Q	+12.4 ± 7.3	153.8	-	-
F3Q	+20.8 ± 4.6	169.8	-	-
F1L	+23.7 ± 7.6	291.6	20	6
F2L	+15.4 ± 7.0	131.3	7	80
F3L	+20.6 ± 4.3	273.9	7	90
Parent	+40.6 ± 4.6	909.1	200	0

^1^ FiQ: Fractions obtained by depolymerization with chitosanase; FiL: Fractions obtained by depolymerization with lysozyme. ^2^ Data determined from dynamic light scattering measurement. ^3^ Data determined from transmission electron microscopy images.
